# The effect of a night shift nap on post-night shift performance, sleepiness, mood, and first recovery sleep: A randomized crossover trial

**DOI:** 10.5271/sjweh.4129

**Published:** 2024-01-01

**Authors:** P Daniel Patterson, Cassie J Hilditch, Matthew D Weaver, David GL Roach, Tiffany S Okerman, Sarah E Martin, Charity G Patterson, Leonard S Weiss

**Affiliations:** 1 University of Pittsburgh, School of Medicine, Department of Emergency Medicine, Pittsburgh, PA, USA.; 2 University of Pittsburgh, School of Health and Rehabilitation Sciences, Department of Community Health Services and Rehabilitation Sciences, Emergency Medicine Program, Pittsburgh, PA, USA.; 3 San José State University, Fatigue Countermeasures Laboratory, San José, CA, USA.; 4 Brigham and Women’s Hospital, Division of Sleep and Circadian Disorders, Boston, MA, USA.; 5 Harvard Medical School, Division of Sleep Medicine, Boston, MA, USA.; 6 University of Pittsburgh, School of Health and Rehabilitation Sciences Data Center, Pittsburgh, PA,USA.

**Keywords:** napping, shift work, shift worker

## Abstract

**Objectives:**

This study aimed to test the effect of a 30-minute nap versus a 2-hour nap opportunity taken during a simulated night shift on performance, fatigue, sleepiness, mood, and sleep at the end of shift and during post-night shift recovery.

**Methods:**

We conducted a randomized crossover trial of three nap conditions (30-minute, 2-hour, and no-nap) during 12-hour simulated night shifts. We tested for differences in performance, fatigue, sleepiness, mood, and sleep during in-lab and at-home recovery. Performance was measured with the Brief Psychomotor Vigilance Test (PVT-B). Subjective ratings were assessed with single-item surveys.

**Results:**

Twenty-eight individuals consented to participate [mean age 24.4 (standard deviation 7.2) years; 53.6% female; 85.7% Emergency Medical Services clinicians]. PVT-B false starts at the end of the 12-hour night shift (at 07:00 hours) and at the start of in-lab recovery (08:00 hours) were lower following the 2-hour nap versus other conditions (P<0.05). PVT-B response time at +0 minutes post-recovery nap was poorer compared to pre-recovery nap for the no-nap condition (P=0.003), yet not detected for other nap conditions (P>0.05). Sleepiness, fatigue, and some mood states were lower at most hourly assessments during the in-lab recovery period following the 2-hour nap condition compared to the other conditions. Sleep during recovery did not differ by duration of night shift nap.

**Conclusions:**

A 2-hour nap opportunity versus a 30-minute or no-nap opportunity is beneficial for performance, alertness, and mood post-night shift. No differences were detected in sleep during recovery.

Night shift work is associated with sleep loss, fatigue, poor mood states, poorer health, and impaired vigilance during and after work hours (1–4). Many shift workers report inadequate recovery between scheduled shifts (5), which raises questions about effective on-shift interventions that may mitigate the negative effects of night shift work and improve inter-shift recovery. Napping on-duty is supported by the best available evidence as well as leading sleep medicine organizations (6–8). One study suggests that an extended on-duty nap of 2 hours may mitigate the negative effects of night shift work (9). However, the efficacy of a 2-hour nap during night shift work versus naps of other durations has not been adequately tested (10). In addition, employer support for a 2-hour nap during night shift work may be limited given that long duration naps may impede work or productivity, increase the risk of and severity of sleep inertia, and may not have adequate support from all stakeholders (11).

In this secondary analysis of a randomized crossover trial (12), we test the effect of a 30-minute versus 2-hour nap opportunity taken during a simulated night shift on cognitive performance, fatigue, sleepiness, mood states, and sleep at the end of shift and throughout the immediate post-night shift recovery opportunity. We focus on the post-night shift recovery opportunity given that many night shift workers, especially in public safety and certain healthcare settings, may have the opportunity for longer naps during night shift work (ie, 2 hours) (13) and may subsequently experience delays in obtaining sleep post-night shift due to the required commute home and non-work related responsibilities that can only be completed during daylight hours (eg, childcare, running errands). The post-night shift period (recovery opportunity) is also a period of safety concern for employers and employees given evidence of elevated sleepiness and risk of motor vehicle crashes during the commute home (14–17). Our study and focus are unique, yet the findings are generalizable to many night shift workers and employers concerned with the recovery opportunity after night shift work or between scheduled shifts.

## Methods

### General study design

We conducted a randomized crossover trial with participants assigned to three separate 72-hour conditions comprised of 36 hours of at-home monitoring (07:00–19:00 + 1 day), followed by a 12-hour in-lab night shift (19:00–07:00), 12-hour in-lab recovery opportunity (07:00–19:00), and 12-hour at-home recovery opportunity (19:00–07:00) (12). Two intervention conditions included a nap that began at 02:00 (30 minutes or 2 hours) during the simulated night shift. The third condition was a no-nap control. The order of conditions was randomized. Participants were given a 2-hour daytime recovery nap opportunity (beginning at 13:00) during the in-lab recovery opportunity period (standard across all conditions). Participants concluded each condition with 12 hours of at-home recovery opportunity that included a prescribed sleep opportunity from 22:00–07:00.

### Study setting and participants

Our participants were recruited from generally healthy public safety workers certified as Emergency Medical Technicians (EMT), paramedics, nurses, or other healthcare clinicians residing in Western Pennsylvania, USA.

### Study protocol and measures

Participants completed a baseline survey with reliable and valid questionnaires (eg, the Pittsburgh Sleep Quality Index, PSQI) (18), a paper sleep diary during at-home periods, and answered a battery of single-item survey questions to assess subjective ratings of sleepiness, fatigue, and mood at hourly intervals (19). This battery has been used previously and demonstrated significant correlations with established measures of sleep quality (19). Nap-specific measures were assessed immediately pre-nap and again at +0, +10, +20, and +30 minutes post-nap. Participants wore multiple non-invasive physiological devices throughout each condition (findings reported elsewhere) (12, 20) and completed the Brief 3-minute version of the Psychomotor Vigilance Test (PVT-B) (21). The PVT-B measured reaction time [RT in milliseconds (ms)], lapses (RT>355ms), false starts (reactions before stimulus or RT<100ms), and speed (1000/RT) (21). Participants wore wrist actigraphy continuously during each condition and were fitted with portable electroencephalogram (EEG) during scheduled in-lab naps to assess sleep architecture. We used the wGT3X-BT wrist-worn actigraphy device (Actigraph Corporation, Pensacolo, Florida, USA) and the Zmachine® Synergy portable EEG device (General Sleep Corporation, Cleveland, Ohio, USA). The devices have been used in multiple observational and experimental studies to monitor sleep and wake (22, 23). During the simulated night shift, participants were permitted to read, watch television, and use the computer. We addressed ecological validity of our protocol by administering to all participants, at random, four low-fidelity mock patient care scenarios (two prior to 02:00 and two prior to 07:00). Separate publications report details of our protocol and findings for other outcomes of interest (12, 20, 24).

### Statistical analysis

This analysis reports on the post-night shift in-lab recovery opportunity period (07:00–19:00) and final at-home recovery opportunity period (19:00–07:00). We used descriptive statistics to characterize our study sample (eg, frequencies and means). Given the crossover study design, we used linear mixed-effects models that accounted for the dependence between repeated subject assessments to compare PVT-B outcomes at various time points and to test for differences at various time points in the battery of subjective ratings taken each hour during the in lab recovery opportunity period. We also used linear mixed models to compare pre-recovery nap opportunity measures with post-recovery nap measures at +0, +10, +20, +30 minutes (the pre-post nap delta). Given the number of comparisons, we examined Bonferroni corrected P-values. In select comparisons, we excluded data from three participants who deviated from protocol during one of the three conditions. All analyses were performed with the SAS software Version 9.4 (Cary, North Carolina). The original sample size and goal enrollment of 35 was derived to account for an expected attrition of 10 and to detect a difference of 5mmHg in blood pressure between nap conditions (12). Complete data from 25 participants provided 80% power to detect group differences (12).

## Results

Of the 58 individuals who were screened, 28 consented to participate, 1 withdrew from the study and 1 was lost to follow-up prior to completing the protocol. The mean age of participants was 24.4 (standard deviation 7.2) years. Most were female (53.6%), certified as an EMT or paramedic (85.7%), and mostly working 12-hour shifts (53.6%). All three 72-hour conditions were completed by 26 individuals, and 3 deviated from protocol during one of the three conditions.

### PVT-B outcomes

**Figure 1-A and 1-B:**
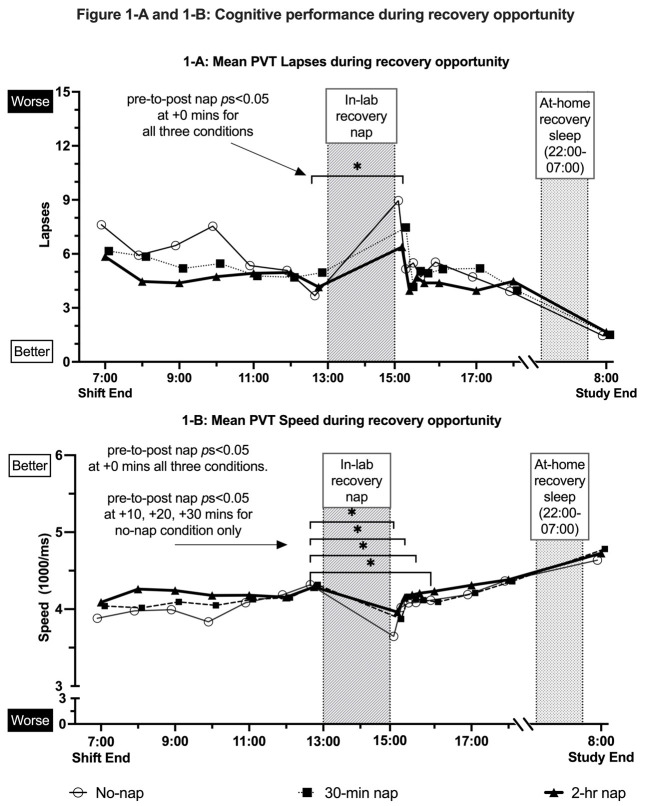
Cognitive performance during recovery opportunity. 1-A reports mean PVT lapses during recovery opportunity. 1-B reports mean PVT speed during recovery opportunity. In addition to hourly point estimates, graphs report the mean difference (Delta) from pre-nap to post-nap at +0, +10, +20, and +30 minutess. Whiskers reporting standard deviation for each point estimate (mean) are not shown to improve interpretation of point estimates and trend over time. Differences by nap condition assessed with linear mixed-effects models that accounted for the dependence between repeated subject assessments. Pairwise comparisons by nap condition assessed based Bonferroni corrected P-values. The * asterisk at hourly assessments indicates Bonferroni P<0.05.

PVT-B false starts at the end of the 12-hour night shift (at 07:00) and at the start of the in-lab recovery opportunity phase (08:00) were lower following the 2-hour night shift nap condition versus the 30-minute night shift nap and no-nap conditions (P<0.05; supplementary material, www.sjweh.fi/article/4129, figure S-2). No other differences by nap condition were detected at hourly assessments during the in-lab recovery opportunity period 09:00–12:00 (figures 1-A and 1-B and supplementary figures S-1 and S-2). For all three conditions, performance immediately after the in-lab recovery nap at +0 minutes was poorer compared to pre-recovery nap for PVT-B lapses and PVT-B speed (P<0.05; figure 1-A, 1-B, and supplementary figures S-1 and S-2). PVT-B RT at +0 minutes post-recovery nap was poorer compared to pre-recovery nap for the no-nap night shift condition (P=0.003; supplementary figure S-1), yet there were no differences detected for the 30-minute or 2-hour conditions (P>0.05). With the exception of the 30-minute night shift nap condition at +20 minutes post-recovery nap (P=0.015; supplementary figure S-2), none of the three conditions showed differences in post-recovery nap PVT false starts compared to pre-recovery nap at +0, +10, +20, or +30 minutes post-recovery nap (P>0.05). Slower PVT-B speed persisted at +10, +20, and +30 minutes post-recovery nap for the no-nap condition (P<0.05; figure 1-B); yet there were no differences detected at these time points compared to pre-nap for the 30-minute or 2-hour night shift nap conditions (P>0.05).

### Subjective scales

Participants self-reported less sleepiness, fatigue, difficulty with concentration, and exhaustion at most – but not all – hourly assessments during the in-lab recovery opportunity period at 08:00–12:00 following the 2-hour night shift nap condition compared to the 30-minute and no-nap conditions (supplementary figure S-3 to S-16). Participants also reported lower levels of sadness at the end of the simulated night shift (at 07:00) and feeling more energetic at 10:00 during the in-lab daytime recovery phase following the 2-hour night shift nap versus the other nap conditions (P<0.05; supplementary figures S-11 and S-12). After completing the no-nap night shift condition, participants reported feeling less sleepiness, less fatigued, more efficient, and less exhausted at +0, +10, +20, and +30 minutes immediately following in the in-lab recovery nap compared to pre-nap measures (P<0.05; supplementary figures S-3, S-4, S-15, and S-16). Similar improvements in sleepiness, fatigue, and exhaustion were reported for the 30-minute and 2-hour night shift nap conditions when comparing pre-to-post recovery nap at +10, +20, and +30 minutes post-nap (P<0.05). Improvements in feeling efficient were reported at all post-recovery nap time points in the no-nap night shift condition only (P<0.05; supplementary figure S-15). Participants reported less difficulty with concentration and improved alertness at +10, +20, and +30 minutes following the in-lab recovery nap when completing the 30-minute and 2-hour night shift nap conditions, but not when completing the no-nap night shift condition (P<0.05; supplementary figures S-5 and S-6). Participants reported no changes in feeling stressed, relaxed, tense, sad, happy, or irritable from pre- to +0, +10, +20, and +30 minutes post-recovery nap for any of the three night shift nap conditions (P>0.05; supplementary figures S-8, S-9, S-10, S-11, S-13, and S-14).

### Sleep

No differences were detected in total sleep, total deep sleep, and sleep efficiency during the in-lab daytime recovery nap among night shift nap conditions (P>0.05; table 1). The total minutes of sleep obtained during the final 9-hour at-home recovery sleep opportunity did not differ between conditions (P>0.05).

**Table 1:** Sleep parameters during daytime in-lab recovery nap opportunity and at-home recovery period. Comparison of in-lab total sleep time (TST) by nap condition P=0.8160. Comparison of sleep efficiency by nap condition P=0.8341. Comparison of light sleep by nap condition P=0.6022. Comparison of deep sleep by nap condition P=0.3181. Comparison of REM sleep by nap condition P=0.6682. Comparison of at-home TST by nap condition P=0.6061.

Variable	No-nap condition					30-minute nap condition					2-hour nap condition			
	In-lab recovery nap (EEG) ^a^			At-home recovery sleep (ACT) ^b^		In-lab recovery nap (EEG) ^a^			At-home recovery sleep (ACT) ^b^		In-lab recovery nap (EEG) ^a^			At-home recovery sleep (ACT) ^b^
	Mean (SD)	% (SD)		Mean (SD)		Mean (SD)	% (SD)		Mean (SD)		Mean (SD)	% (SD)		Mean (SD)
TST (min)	92.6 (15.3)			456.1 (80.1)		93.8 (13.6)			473.7 (89.8)		90.9 (17.2)			451.2 (68.6)
Sleep efficiency		77.1 (12.8)					78.1 (11.6)					75.8 (13.7)		
Light sleep (min)	37.7 (12.9)					41.7 (22.2)					42.8 (16.8)			
Deep sleep (min)	37.6 (18.6)					36.8 (22.2)					29.7 (16.6)			
REM sleep (min)	17.3 (10.5)					15.3 (11.4)					18.4 (12.4)			

^a^ Sleep was assessed with the Zmachine® Synergy portable electroencephalogram device (General Sleep Corp, Cleveland, OH, USA). ^b^ Sleep was assessed with the wrist-worn actigraphy device wGT3X-BT (Actigraph Corp, Pensacola, FL, USA).

## Discussion

Napping during night shift work is recommended and supported by the best available evidence (7, 8), yet many questions remain regarding the positive and negative effects of on-shift napping on key outcomes of interest, especially post-night shift during a period of recovery (10). During this critical period, it is known that sleepiness post-night shift is increased (15, 17), performance is poorer, and risk of negative safety outcomes are elevated following simulated night shift work (17), especially if the worker was unable to obtain a nap during night shift work. Key findings from the current study shows that during the first few hours after a simulated 12-hour night shift (initial post-night shift recovery opportunity), there were fewer deficits in cognitive performance, lower levels of fatigue, sleepiness, and difficulty concentrating when participants were provided a 2-hour versus a 30-minute nap opportunity or no-nap. Our findings are generalizable to night shift workers who experience disruption in their post-night shift schedule or the inability to obtain sleep immediately post-night shift, and must delay their sleep opportunity, due to home-related responsibilities such as transportation home, childcare, running errands, or other tasks that require wakefulness during daylight hours.

Another key finding from our study is that a 2-hour nap during night shifts does not negatively impact key indicators of sleep either during daytime recovery opportunity sleep post-night shift or subsequent nighttime recovery sleep. Our findings conflict with prior studies that report significantly less deep sleep (slow wave) during daytime recovery naps following a simulated night shift with long duration naps (eg, 50 minutes or 2 hours) compared to shorter nap opportunities or no-nap (25, 26). Differences may be attributed to when the recovery nap was initiated and the duration of nap.

While we conclude that a 2-hour on-shift nap opportunity, versus a 30-minute or no-nap opportunity, is beneficial for the time period immediately after a 12-hour night shift (the initial recovery opportunity), we also recognize there are limitations to our findings. First, our protocol was designed to account for the inability to obtain sleep immediately post-night shift. Our study design is unique and informed by experiences often faced by public safety workers and healthcare workers. In these settings, workload can be variable during night shifts and reduced during early morning hours (eg, 02:00–05:00) (27, 28). Employers that allow napping may not specify a maximum duration, given limited guidance on optimal nap duration or other factors (6, 10). As such, the worker may obtain an extended nap of ≥2 hours, which we have observed in previous research (13). The protocol we tested was based on the experiences of these shift workers (and others like them) and, therefore, our findings may not apply to night shift workers in occupations where a 2-hour nap opportunity is not practical. Second, laboratory conditions are tightly controlled and cannot fully replicate real world conditions. Both in-lab nap opportunities during the simulated night shift began at the same time (02:00), which means participants were awake longer during the post-night shift period following the 30-minute versus 2-hour nap. Some of the group differences reported may be attributable to this feature of our protocol. Additional research on nap timing is needed. Third, during the simulated night shift, we attempted to replicate real-world conditions with four brief simulated patient-care scenarios, yet we recognize these scenarios are limited because they were low-fidelity and brief and may not impact the participant in ways associated with real-world work.

### Concluding remarks

The post-night shift period is a safety-sensitive period and important for worker recovery. When the opportunity to sleep after night shift work is delayed or otherwise postponed, our findings suggest that a 2-hour nap opportunity during the night shift is superior to a 30-minute nap opportunity or no-nap for post-night shift performance, sleepiness, fatigue, and mood. Our findings may be useful to employers responsible for shift work scheduling, fatigue risk management, or worker well-being.

## Supplementary material

Supplementary material

## Protection of research participants

The University of Pittsburgh Institutional Review Board approved this study (STUDY19120222), which was registered with ClinicalTrials.gov on 8 July 2020 (NCT04469803). All participants gave informed consent prior to participation.
