# Supplementary material for: The effect of a night shift nap on post-night shift performance, sleepiness, mood, and first recovery sleep: A randomized crossover trial
Source: Scand J Work Environ Health. 2023 Dec 18;50(1):22–7. doi: 10.5271/sjweh.4129 (PMC10924715; doi:10.5271/sjweh.4129)
Supplement: Supplementary material [file SJWEH-50-22-S001.pdf]

The effect of a night shift nap on post-night shift performance, sleepiness, mood, and first recovery sleep: A randomized crossover trial[1]

by P Daniel Patterson, PhD,[2] Cassie J Hilditch, PhD, Matthew D Weaver, PhD, David GL Roach, MPH, Tiffany S Okerman, BS, Sarah E Martin, MPH, Charity G Patterson, PhD, Leonard S Weiss, MD

1. Supplementary material
2. Correspondence to: P. Daniel Patterson, PhD, NRP, University of Pittsburgh, School of Medicine, Department of Emergency Medicine, 3600 Forbes Ave., Iroquois Building, Suite 400A, Pittsburgh, PA 15261. Telephone: 412-864-3830. Email: pdp3@pitt.edu.

Supplemental Figure S-1: Mean PVT Reaction Time during recovery opportunity

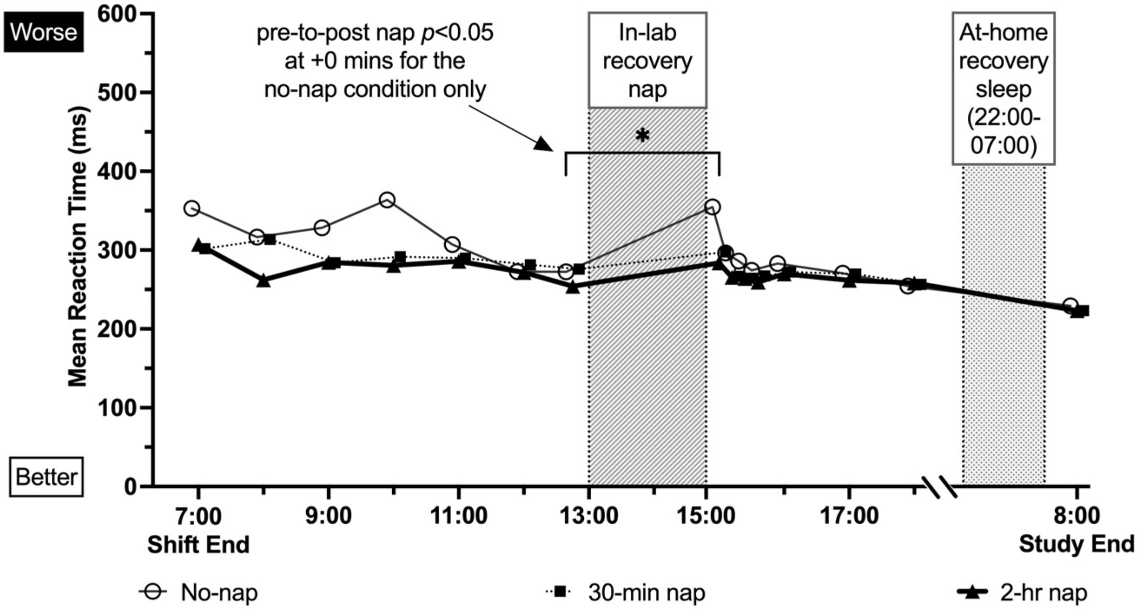

Supplemental Figure S-2: Mean PVT False Starts during recovery opportunity

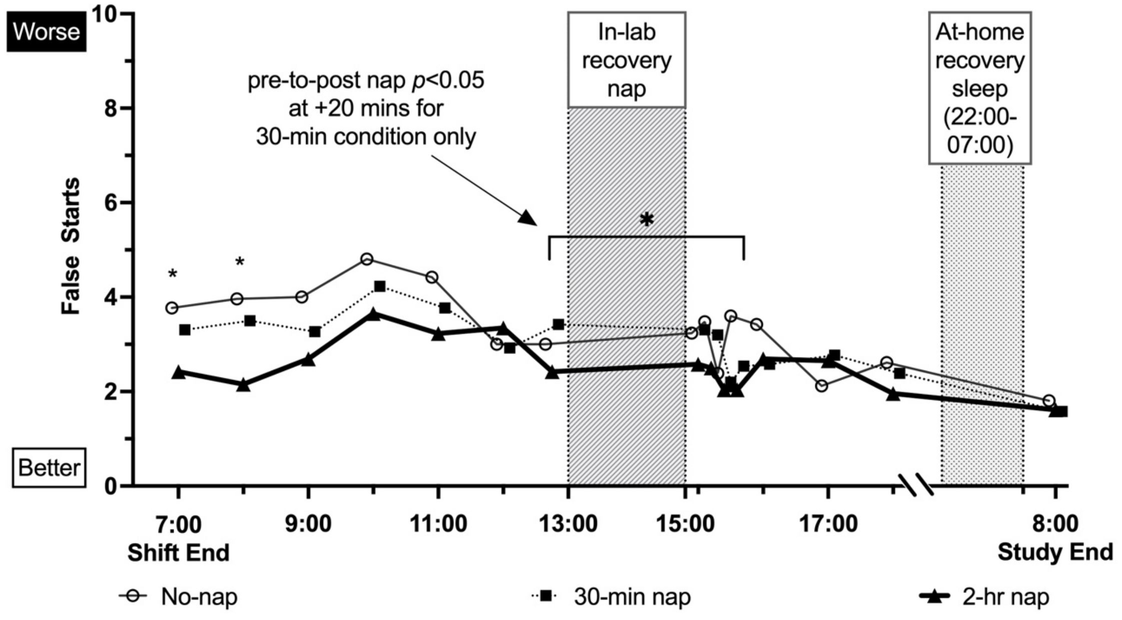

Supplemental Figure S-1 and S-2 footnotes: Supplemental Figure S-1 reports mean PVT reaction time during recovery opportunity. Supplemental Figure S-2 reports mean PVT false starts during recovery opportunity. In addition to hourly point estimates, graphs report the mean difference (Delta) from pre-nap to post-nap at +0, +10, +20, and +30 mins. Whiskers reporting standard deviation for each point estimate (mean) are not shown to improve interpretation of point estimates and trend over time. Differences by nap condition assessed with linear mixed-effects models that accounted for the dependence between repeated subject assessments. Pairwise comparisons by nap condition assessed based Bonferroni corrected p-values. The * asterisk at hourly assessments indicates Bonferroni p-values <0.05.

Supplemental Figure S-3: "Rate your SLEEPINESS right now"

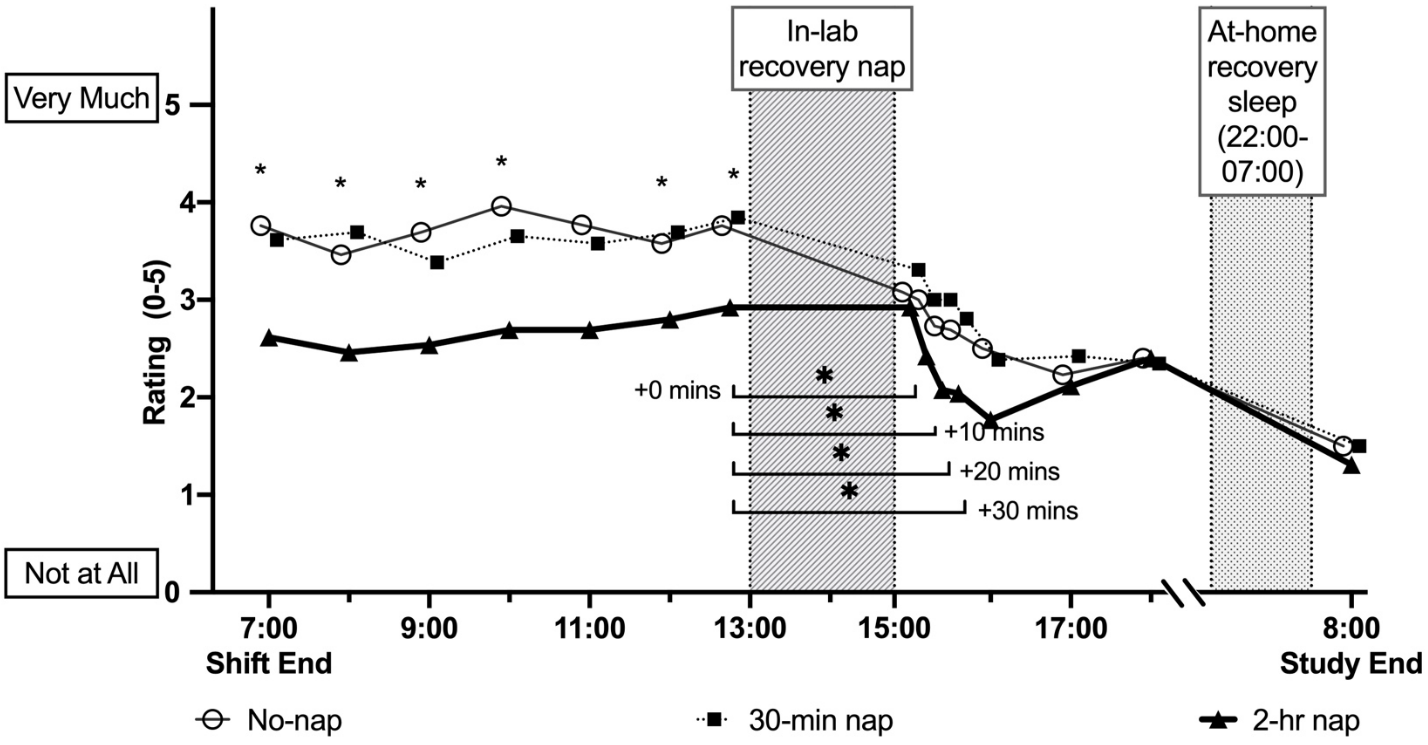

Supplemental Figure S-3 footnotes: This Figure reports means for hourly assessments of SLEEPINESS stratified by nap condition as well as the mean difference (Delta) from pre-nap to post-nap at +0, +10, +20, and +30 mins. Whiskers reporting standard deviation for each point estimate (mean) are not shown to improve interpretation of point estimates and trend over time. Differences by nap condition assessed with linear mixed-effects models that accounted for the dependence between repeated subject assessments. Pairwise comparisons by nap condition assessed based Bonferroni corrected p-values. The * asterisk at hourly assessments indicates Bonferroni p-values <0.05. When compared to pre-nap measures, post-nap measurements for the no-nap condition show less SLEEPINESS at +0, +10, +20, and +30 mins after the in-lab recovery nap opportunity (p-values <0.05). When compared to pre-nap measures, post-nap measurements for the 30-min and 2-hr nap conditions show less SLEEPINESS +10, +20, and +30 mins after the in-lab recovery nap opportunity (p-values <0.05).

Supplemental Figure S-4: “Rate your FATIGUE right now."

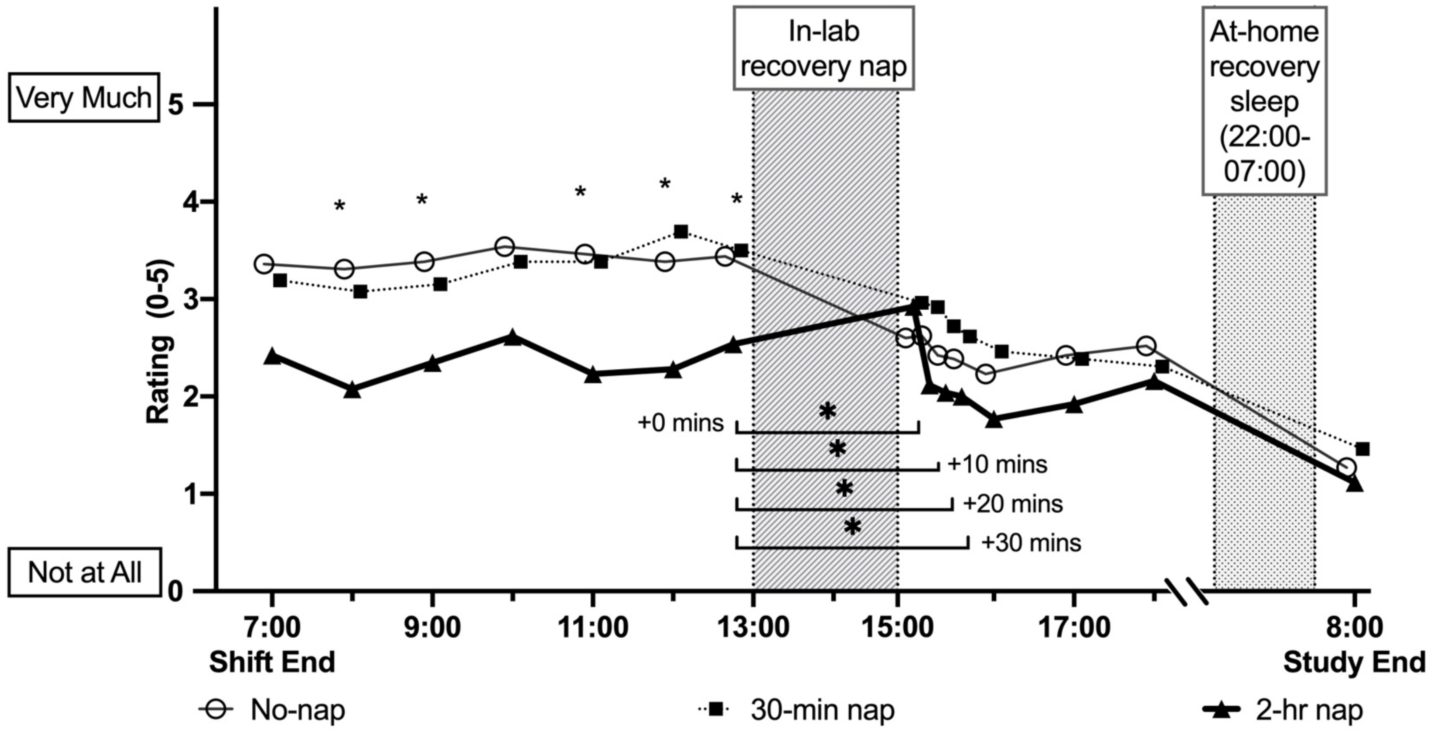

Supplemental Figure S-4 footnotes: This Figure reports means for hourly assessments of FATIGUE stratified by nap condition as well as the mean difference (Delta) from pre-nap to post-nap at +0, +10, +20, and +30 mins. Whiskers reporting standard deviation for each point estimate (mean) are not shown to improve interpretation of point estimates and trend over time. Differences by nap condition assessed with linear mixed-effects models that accounted for the dependence between repeated subject assessments. Pairwise comparisons by nap condition assessed based Bonferroni corrected p-values. The * asterisk at hourly assessments indicates Bonferroni p-values <0.05. When compared to pre-nap measures, post-nap measurements for the no-nap condition show less FATIGUE at +0, +10, +20, and +30 mins after the in-lab recovery nap opportunity (p-values <0.05). When compared to pre-nap measures, post-nap measurements for the 30-min and 2-hr nap conditions show less FATIGUE at +10, +20, and +30 mins after the in-lab recovery nap opportunity (Bonferroni p-values <0.05).

Supplemental Figure S-5: "Rate your difficulty with CONCENTRATION right now"

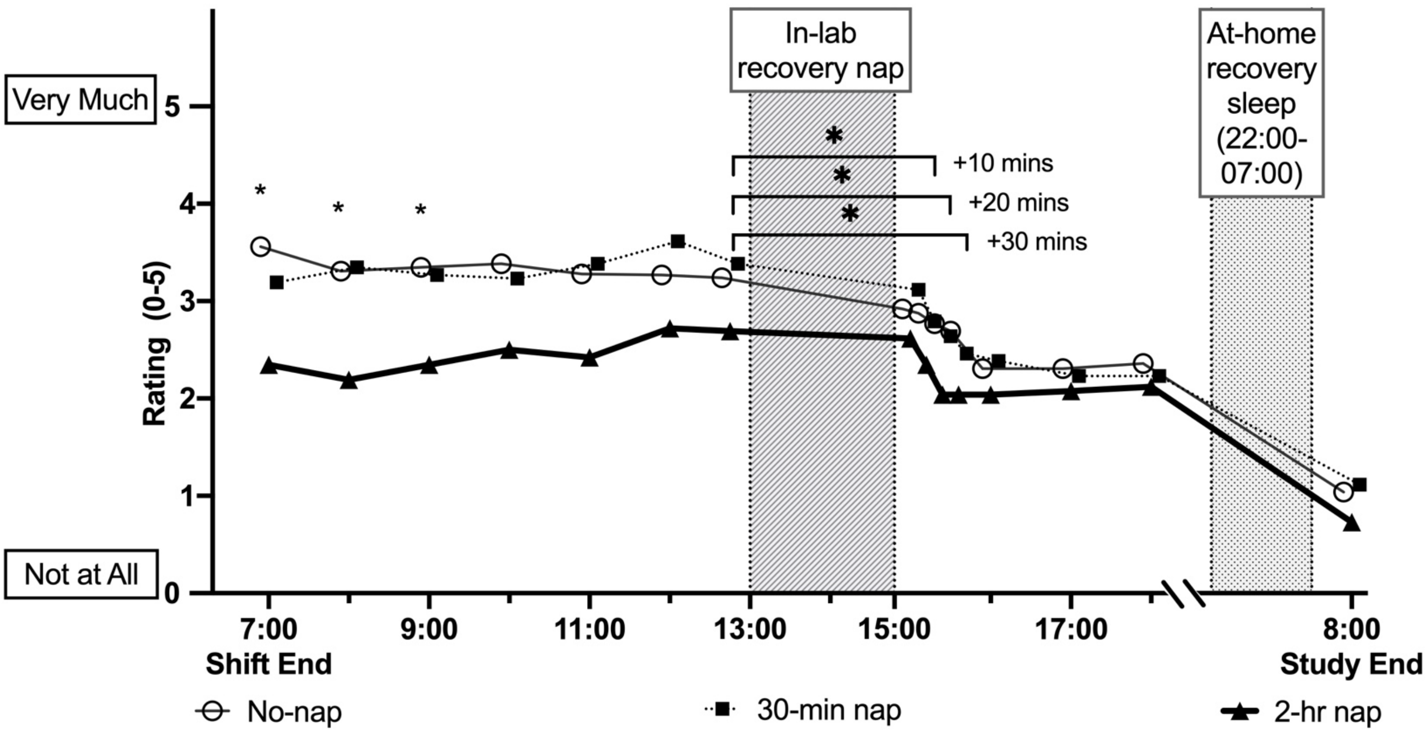

Supplemental Figure S-5 footnotes: This Figure reports means for hourly assessments of DIFFICULTY WITH CONCENTRATION stratified by nap condition as well as the mean difference (Delta) from pre-nap to post-nap at +0, +10, +20, and +30 mins. Whiskers reporting standard deviation for each point estimate (mean) are not shown to improve interpretation of point estimates and trend over time. Differences by nap condition assessed with linear mixed-effects models that accounted for the dependence between repeated subject assessments. Pairwise comparisons by nap condition assessed based Bonferroni corrected p-values. The * asterisk at hourly assessments indicates Bonferroni p-values <0.05. When compared to pre-nap measures, post-nap measurements for the 30-min and 2-hr recovery nap conditions show less DIFFICULTY WITH CONCENTRATION at +10, +20, and +30 mins after the in-lab recovery nap opportunity (p-values <0.05), but not when completing the no-nap condition.

Supplemental Figure S-6: ”How ALERT do you feel right now?"

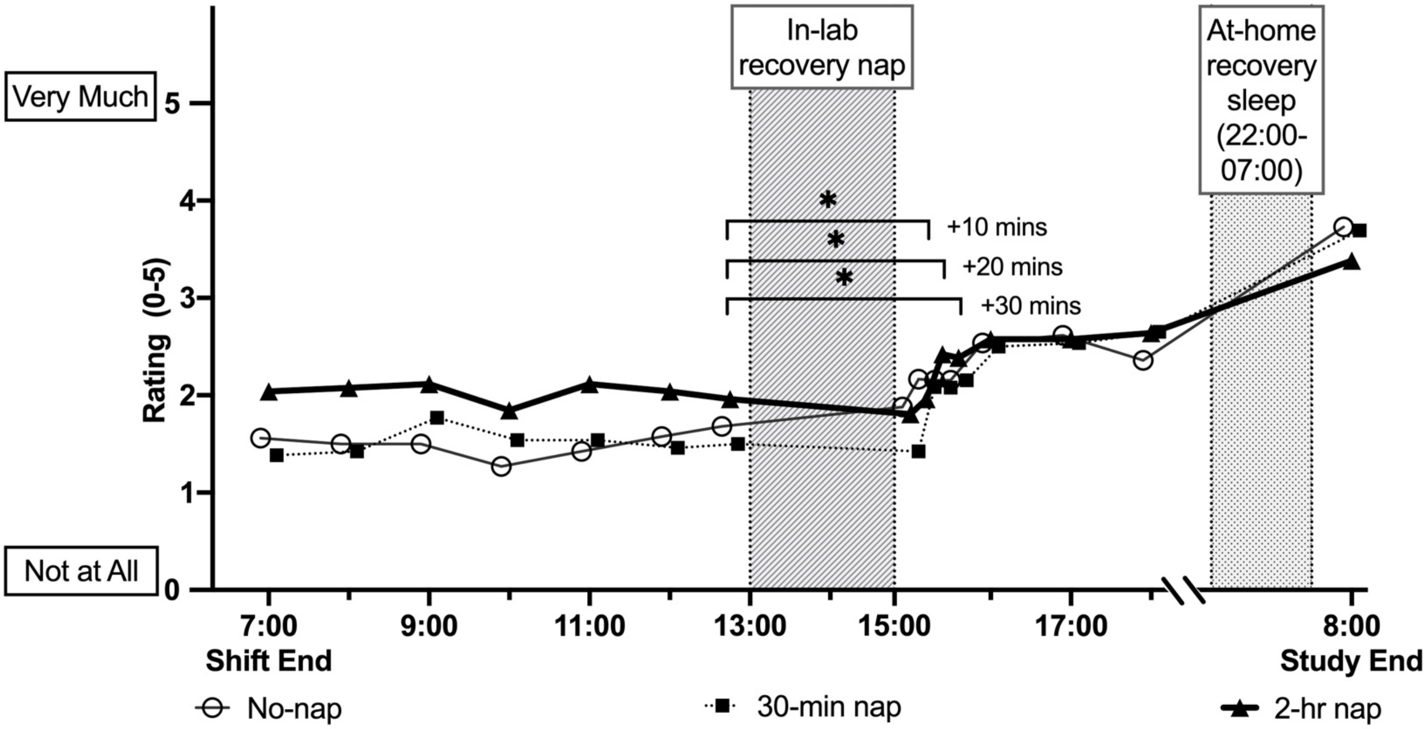

Supplemental Figure S-6 footnotes: This Figure reports means for hourly assessments of ALERTNESS stratified by nap condition as well as the mean difference (Delta) from pre-nap to post-nap at +0, +10, +20, and +30 mins. Whiskers reporting standard deviation for each point estimate (mean) are not shown to improve interpretation of point estimates and trend over time. Differences by nap condition assessed with linear mixed-effects models that accounted for the dependence between repeated subject assessments. Pairwise comparisons by nap condition assessed based Bonferroni corrected p-values. The * asterisk at hourly assessments indicates Bonferroni p-values <0.05. When compared to pre-nap measures, post-nap measurements for the 30-min and 2-hr recovery nap conditions show improved ALERTNESS at +10, +20, and +30 mins after the in-lab recovery nap opportunity (p-values <0.05), but not when completing the no-nap condition.

Supplemental Figure S-7: "How ANXIOUS do you feel right now?"

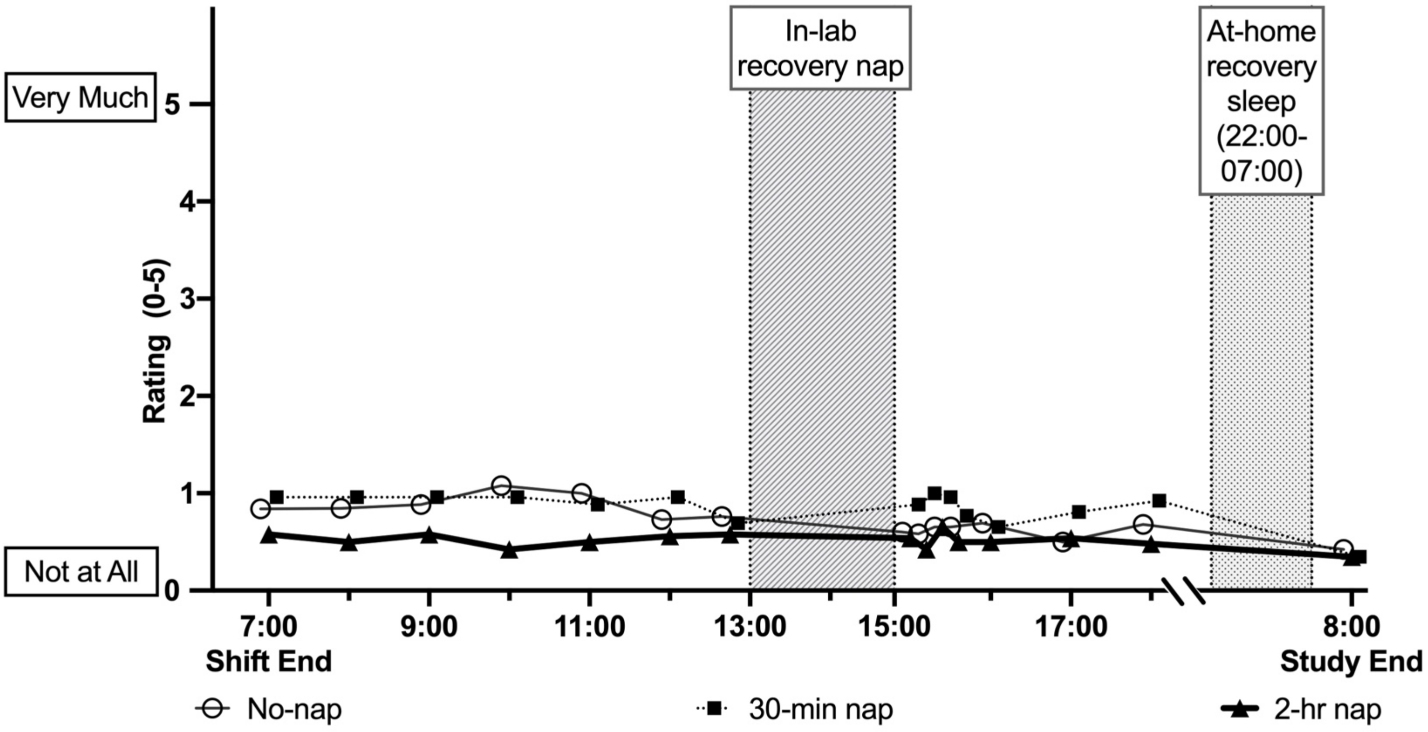

Supplemental Figure S-7 footnotes: This Figure reports means for hourly assessments of ANXIETY stratified by nap condition as well as the mean difference (Delta) from pre-nap to post-nap at +0, +10, +20, and +30 mins. Whiskers reporting standard deviation for each point estimate (mean) are not shown to improve interpretation of point estimates and trend over time. Differences by nap condition assessed with linear mixed-effects models that accounted for the dependence between repeated subject assessments. Pairwise comparisons by nap condition assessed based Bonferroni corrected p-values. The * asterisk at hourly assessments indicates Bonferroni p-values <0.05. No differences detected at hourly assessments or when comparing pre-to-post assessments for the in-lab recovery nap.

Supplemental Figure S-8: ”How STRESSED do you feel right now?"

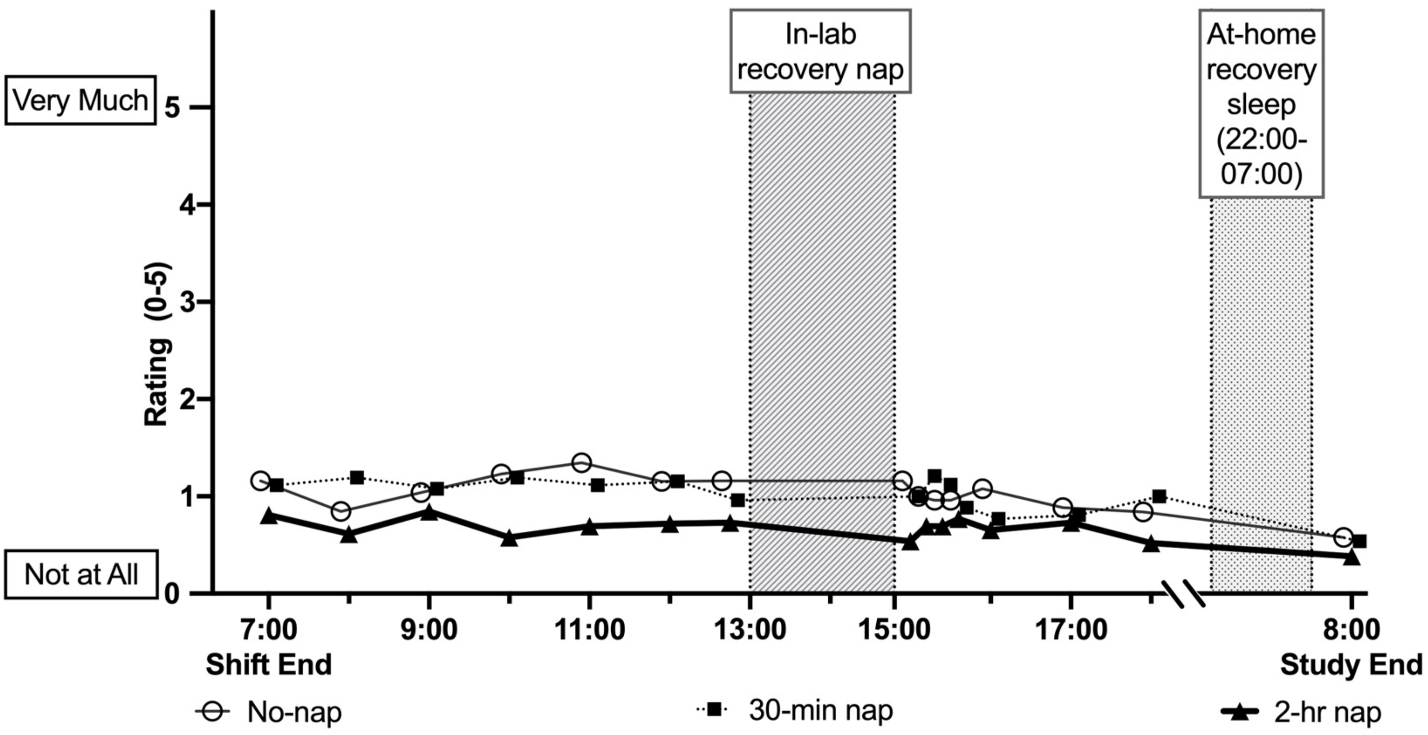

Supplemental Figure S-8 footnotes: This Figure reports means for hourly assessments of STRESS stratified by nap condition as well as the mean difference (Delta) from pre-nap to post-nap at +0, +10, +20, and +30 mins. Whiskers reporting standard deviation for each point estimate (mean) are not shown to improve interpretation of point estimates and trend over time. Differences by nap condition assessed with linear mixed-effects models that accounted for the dependence between repeated subject assessments. Pairwise comparisons by nap condition assessed based Bonferroni corrected p-values. The * asterisk at hourly assessments indicates Bonferroni p-values <0.05. No differences detected at hourly assessments or when comparing pre-to-post assessments for the in-lab recovery nap.

Supplemental Figure S-9: "How RELAXED do you feel right now?"

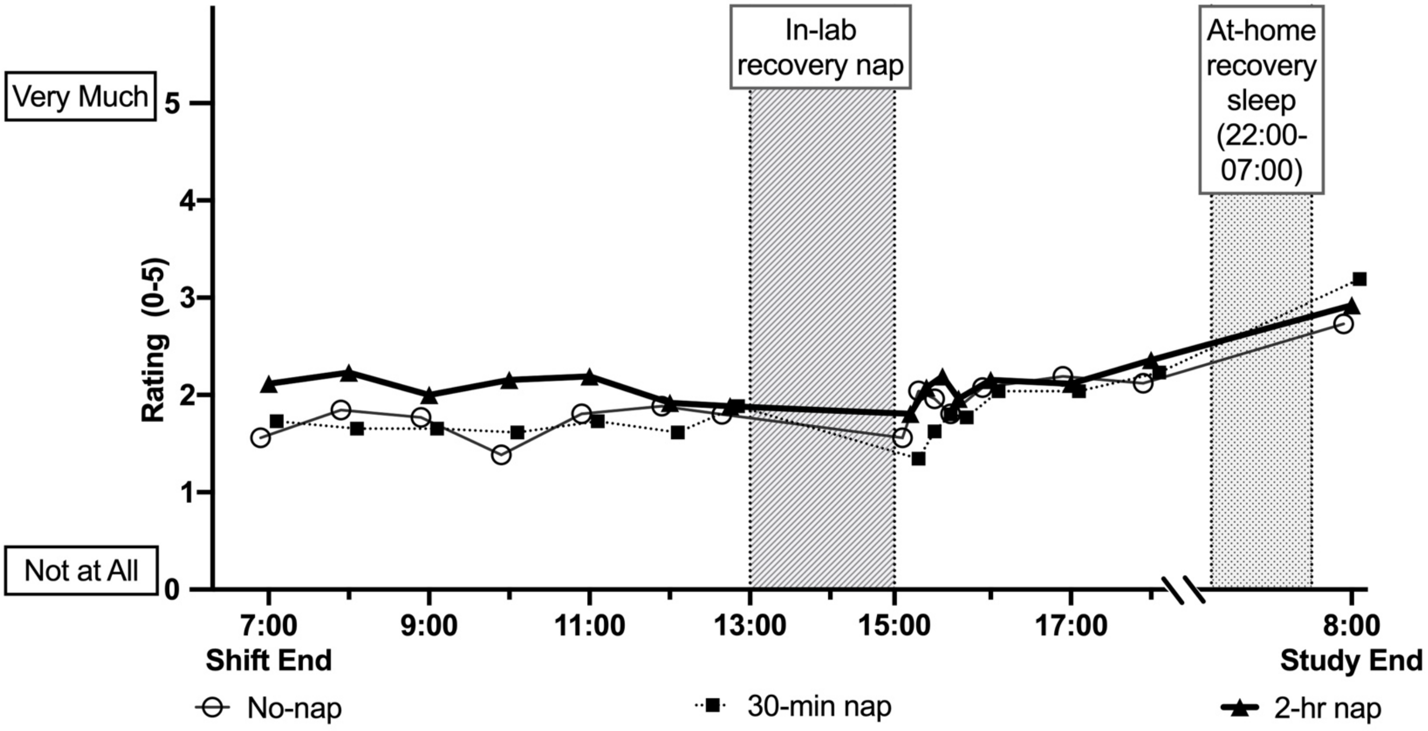

Supplemental Figure S-9 footnotes: This Figure reports means for hourly assessments of RELAXED stratified by nap condition as well as the mean difference (Delta) from pre-nap to post-nap at +0, +10, +20, and +30 mins. Whiskers reporting standard deviation for each point estimate (mean) are not shown to improve interpretation of point estimates and trend over time. Differences by nap condition assessed with linear mixed-effects models that accounted for the dependence between repeated subject assessments. Pairwise comparisons by nap condition assessed based Bonferroni corrected p-values. The * asterisk at hourly assessments indicates Bonferroni p-values <0.05. No differences detected at hourly assessments or when comparing pre-to-post assessments for the in-lab recovery nap.

Supplemental Figure S-10: "How TENSE do you feel right now?"

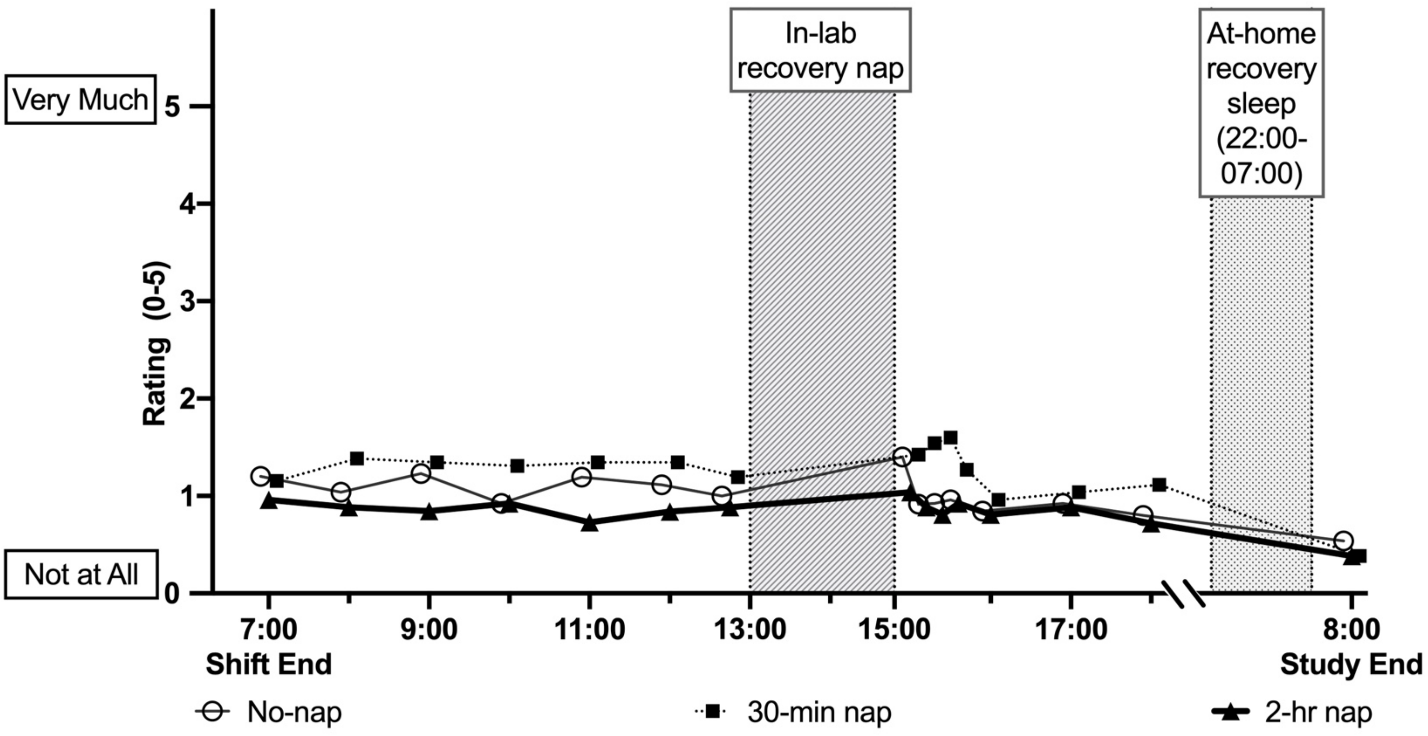

Supplemental Figure S-10 footnotes: This Figure reports means for hourly assessments of TENSE stratified by nap condition as well as the mean difference (Delta) from pre-nap to post-nap at +0, +10, +20, and +30 mins. Whiskers reporting standard deviation for each point estimate (mean) are not shown to improve interpretation of point estimates and trend over time. Differences by nap condition assessed with linear mixed-effects models that accounted for the dependence between repeated subject assessments. Pairwise comparisons by nap condition assessed based Bonferroni corrected p-values. The * asterisk at hourly assessments indicates Bonferroni p-values <0.05. No differences detected at hourly assessments or when comparing pre-to-post assessments for the in-lab recovery nap.

Supplemental Figure S-11: "How SAD do you feel right now?"

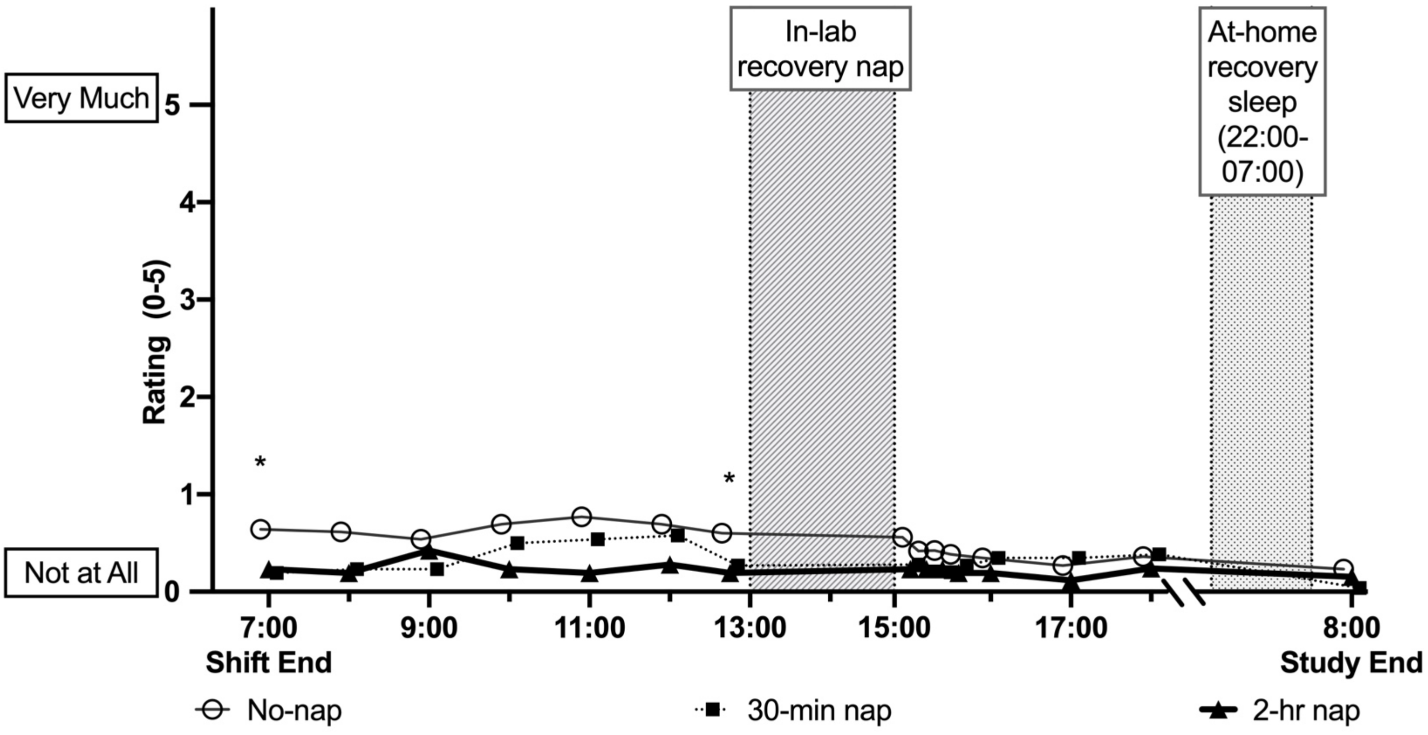

Supplemental Figure S-11 footnotes: This Figure reports means for hourly assessments of SADNESS stratified by nap condition as well as the mean difference (Delta) from pre-nap to post-nap at +0, +10, +20, and +30 mins. Whiskers reporting standard deviation for each point estimate (mean) are not shown to improve interpretation of point estimates and trend over time. Differences by nap condition assessed with linear mixed-effects models that accounted for the dependence between repeated subject assessments. Pairwise comparisons by nap condition assessed based Bonferroni corrected p-values. The * asterisk at hourly assessments indicates Bonferroni p-values <0.05. At 07:00 and immediately before the in-lab recovery nap opportunity (at ~13:00) reports of SADNESS were lower following the 2-hr night shift nap condition versus the other nap conditions (Bonferroni p-value <0.05).

Supplemental Figure S-12: "How ENERGETIC do you feel right now?"

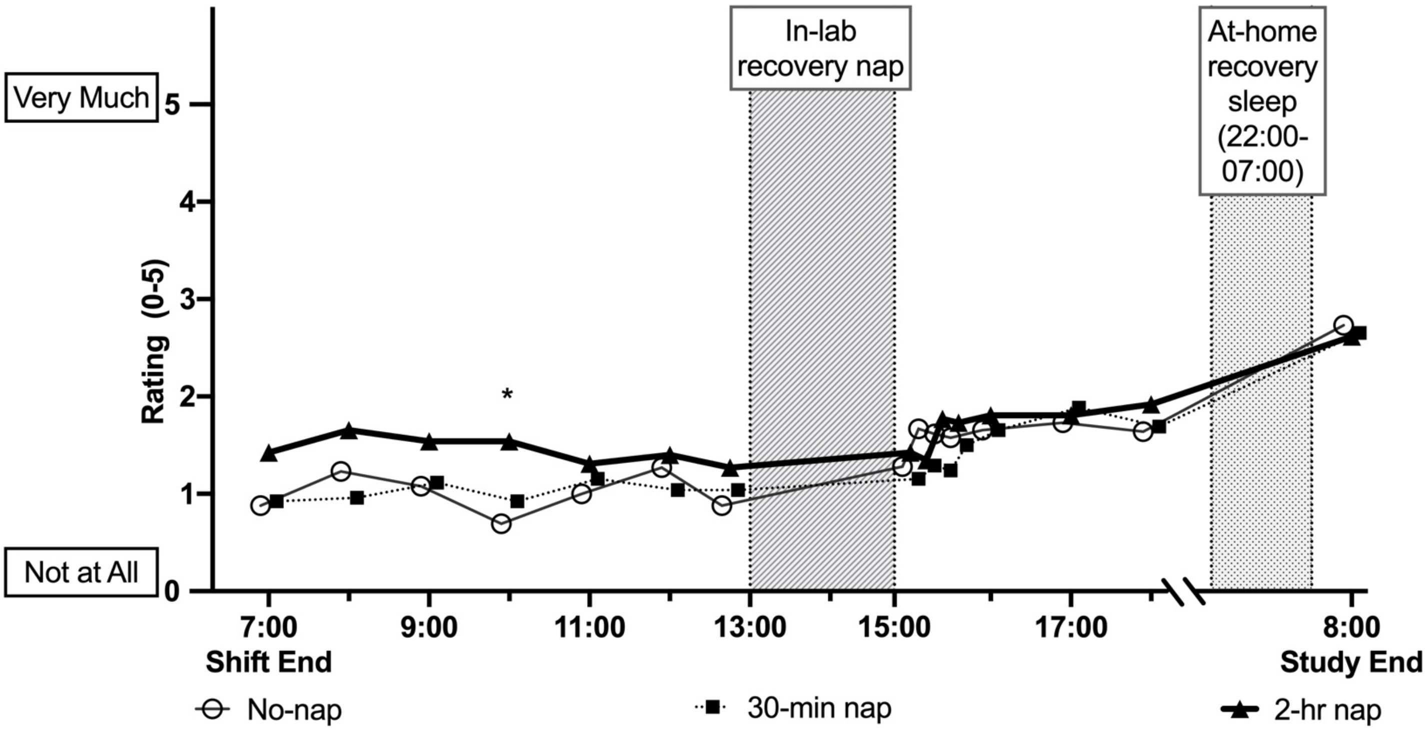

Supplemental Figure S-12 footnotes: This Figure reports means for hourly assessments of feeling ENERGY stratified by nap condition as well as the mean difference (Delta) from pre-nap to post-nap at +0, +10, +20, and +30 mins. Whiskers reporting standard deviation for each point estimate (mean) are not shown to improve interpretation of point estimates and trend over time. Differences by nap condition assessed with linear mixed-effects models that accounted for the dependence between repeated subject assessments. Pairwise comparisons by nap condition assessed based Bonferroni corrected p-values. The * asterisk at hourly assessments indicates Bonferroni p-values <0.05. At 10:00 reports of feeling ENERGETIC were higher following the 2-hr night shift nap condition versus the other nap conditions (Bonferroni p-value <0.05).

Supplemental Figure S-13: "How HAPPY do you feel right now?"

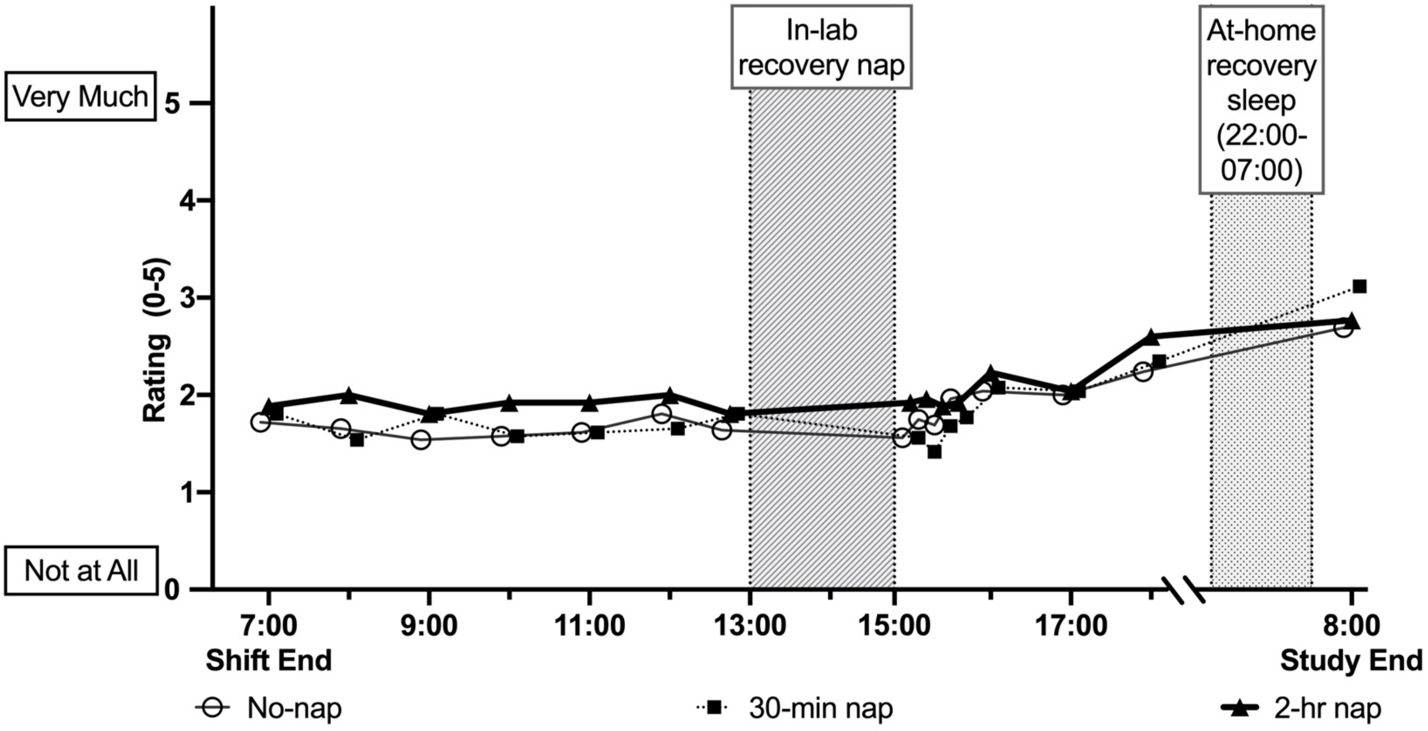

Supplemental Figure S-13 footnotes: This Figure reports means for hourly assessments of feeling HAPPY stratified by nap condition as well as the mean difference (Delta) from pre-nap to post-nap at +0, +10, +20, and +30 mins. Whiskers reporting standard deviation for each point estimate (mean) are not shown to improve interpretation of point estimates and trend over time. Differences by nap condition assessed with linear mixed-effects models that accounted for the dependence between repeated subject assessments. Pairwise comparisons by nap condition assessed based Bonferroni corrected p-values. The * asterisk at hourly assessments indicates Bonferroni p-values <0.05. No differences detected at hourly assessments or when comparing pre-to-post assessments for the in-lab recovery nap.

Supplemental Figure S-14: "How IRRITABLE do you feel right now?"

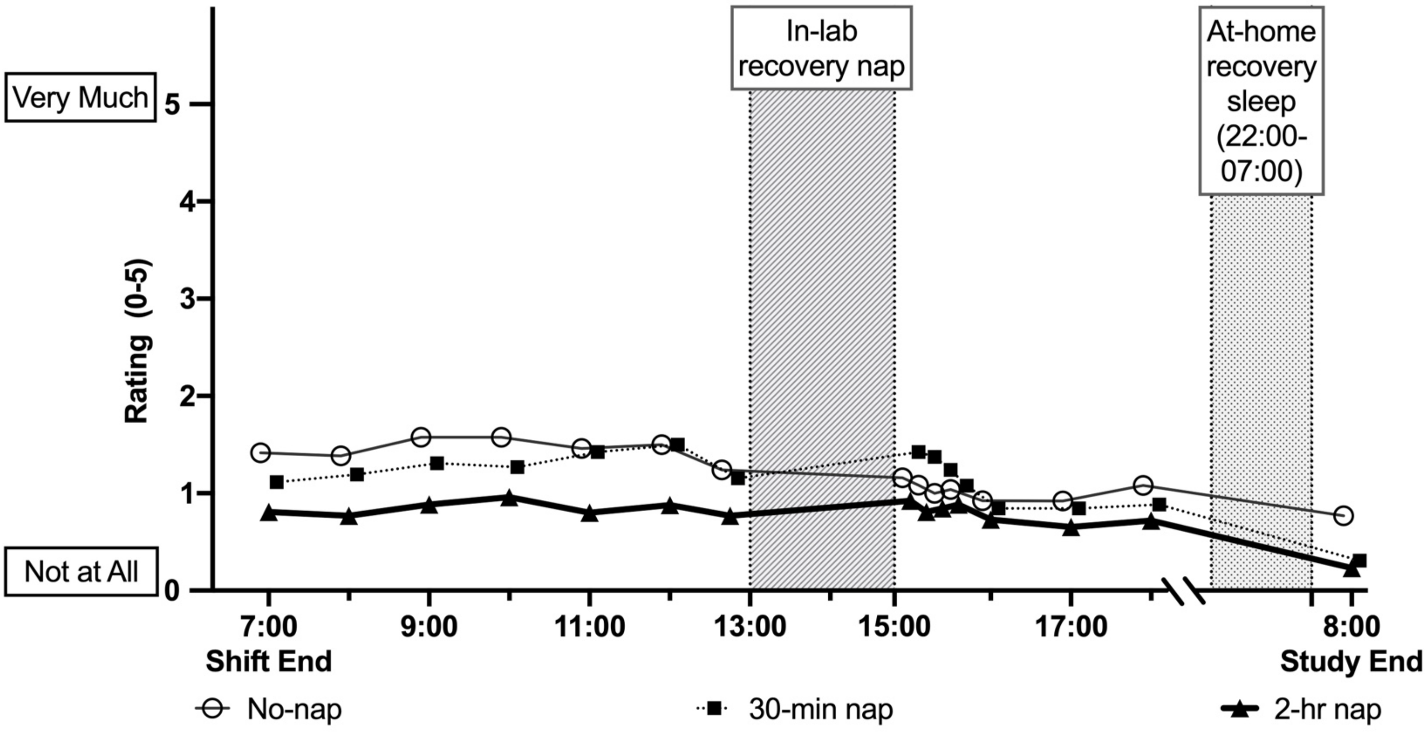

Supplemental Figure S-14 footnotes: This Figure reports means for hourly assessments of feeling IRRITABLE stratified by nap condition as well as the mean difference (Delta) from pre-nap to post-nap at +0, +10, +20, and +30 mins. Whiskers reporting standard deviation for each point estimate (mean) are not shown to improve interpretation of point estimates and trend over time. Differences by nap condition assessed with linear mixed-effects models that accounted for the dependence between repeated subject assessments. Pairwise comparisons by nap condition assessed based Bonferroni corrected p-values. The * asterisk at hourly assessments indicates Bonferroni p-values <0.05. No differences detected at hourly assessments or when comparing pre-to-post assessments for the in-lab recovery nap.

Supplemental Figure S-15: "How EFFICIENT do you feel right now?"

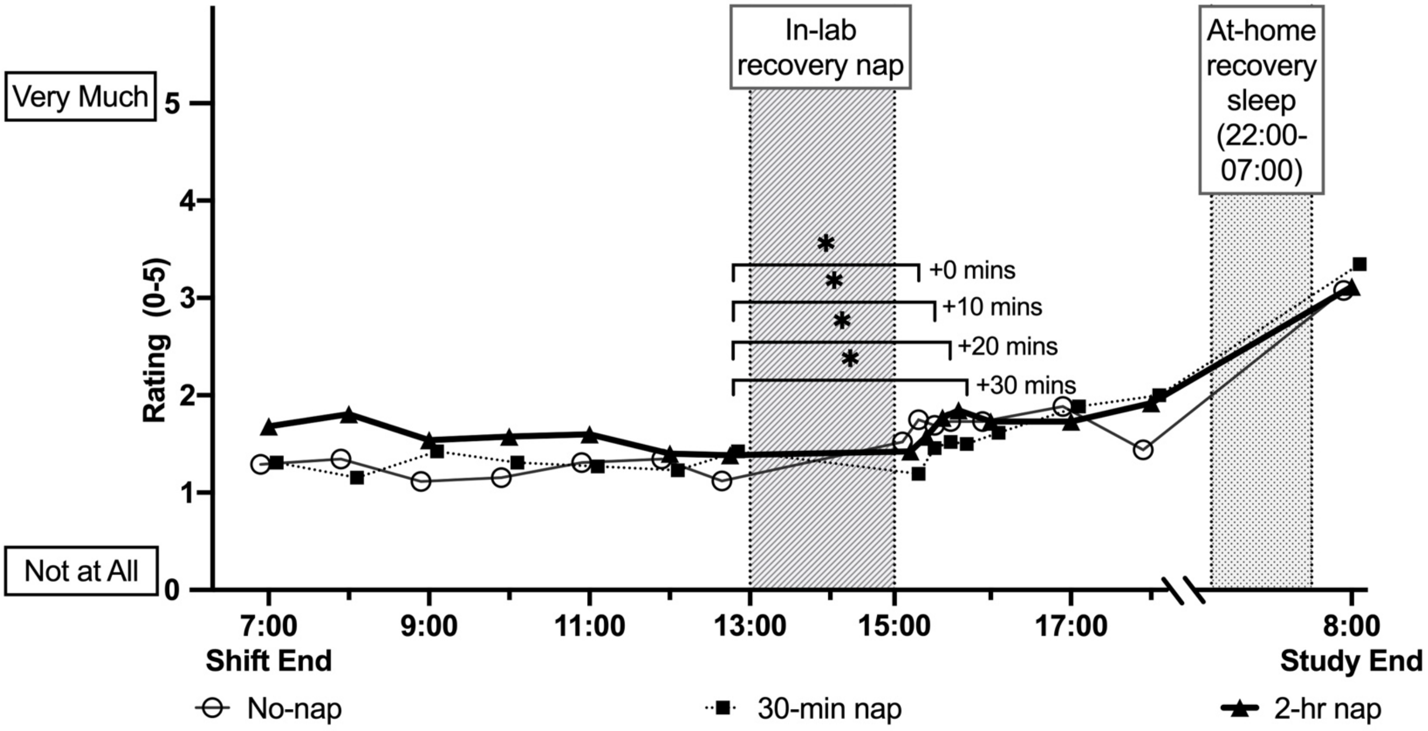

Supplemental Figure S-15 footnotes: This Figure reports means for hourly assessments of feeling EFFICIENT stratified by nap condition as well as the mean difference (Delta) from pre-nap to post-nap at +0, +10, +20, and +30 mins. Whiskers reporting standard deviation for each point estimate (mean) are not shown to improve interpretation of point estimates and trend over time. Differences by nap condition assessed with linear mixed-effects models that accounted for the dependence between repeated subject assessments. Pairwise comparisons by nap condition assessed based Bonferroni corrected p-values. The * asterisk at hourly assessments indicates Bonferroni p-values <0.05. When compared to pre-nap measures, post-nap measurements for the no-nap condition show less EFFICIENT at +0, +10, +20, and +30 mins after the in-lab recovery nap opportunity (p-values <0.05).

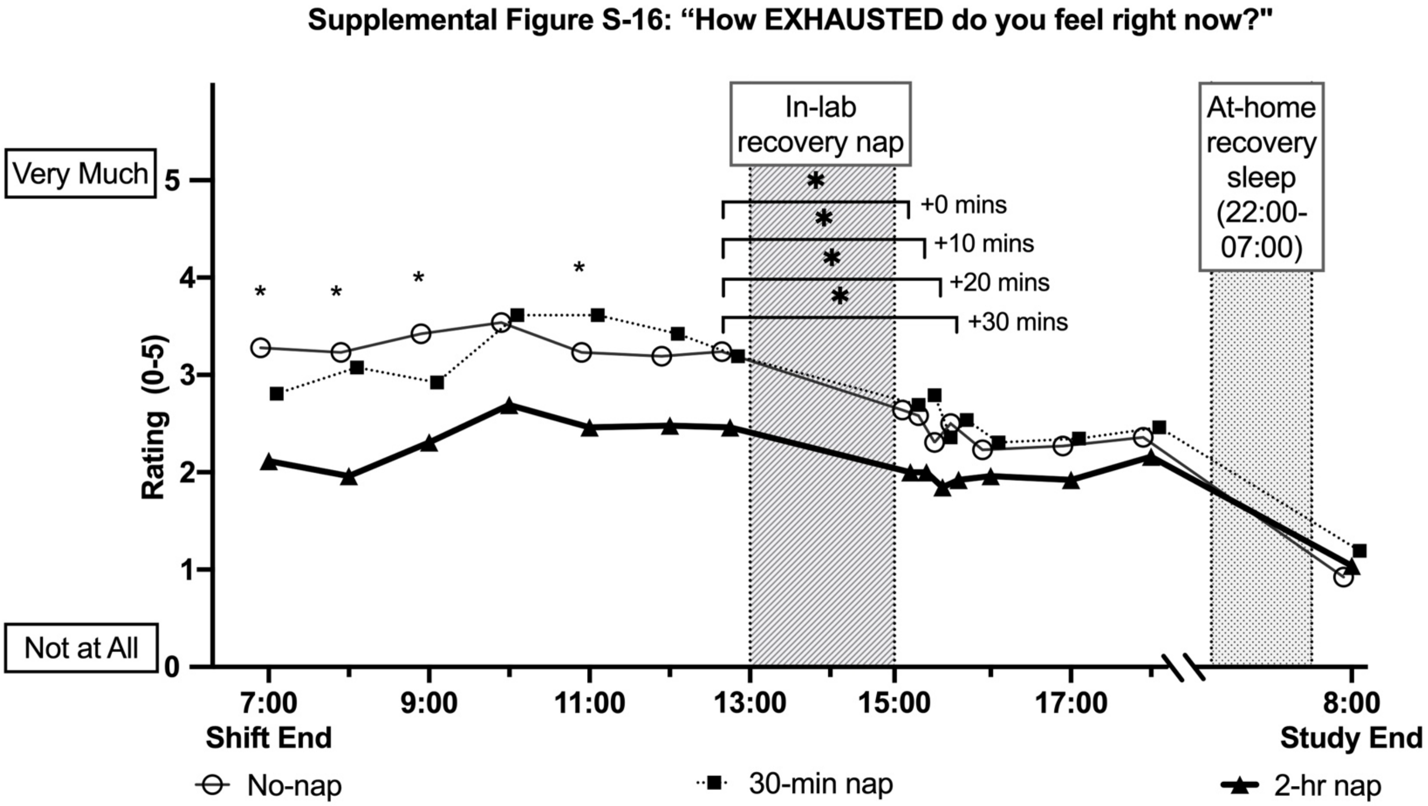

Supplemental Figure S-16 footnotes: This Figure reports means for hourly assessments of feeling EXHAUSTED stratified by nap condition as well as the mean difference (Delta) from pre-nap to post-nap at +0, +10, +20, and +30 mins. Whiskers reporting standard deviation for each point estimate (mean) are not shown to improve interpretation of point estimates and trend over time. Differences by nap condition assessed with linear mixed-effects models that accounted for the dependence between repeated subject assessments. Pairwise comparisons by nap condition assessed based Bonferroni corrected p-values. The * asterisk at hourly assessments indicates Bonferroni p-values <0.05. When compared to pre-nap measures, post-nap measurements for the no-nap condition show less EXHAUSTION at +0, +10, +20, and +30 mins after the in-lab recovery nap opportunity (p-values <0.05). When compared to pre-nap measures, post-nap measurements for the 30-min and 2-hr nap conditions show less EXHAUSTION at +10, +20, and +30 mins after the in-lab recovery nap opportunity (p-values <0.05).
